# The down-regulated ING5 expression in lung cancer: A potential target of gene therapy

**DOI:** 10.18632/oncotarget.10519

**Published:** 2016-07-09

**Authors:** Shuang Zhao, Xue-feng Yang, Dao-fu Shen, Yang Gao, Shuai Shi, Ji-cheng Wu, Hong-xu Liu, Hong-zhi Sun, Rong-jian Su, Hua-chuan Zheng

**Affiliations:** ^1^ Cancer Center, Key Laboratory of Brain and Spinal Cord Injury of Liaoning Province, and Animal Center, The First Affiliated Hospital of Jinzhou Medical University, Jinzhou, 121001, China; ^2^ Department of Thoracic Surgery, The First Affiliated Hospital of China Medical University, Shenyang, 110001, China; ^3^ Life Science Institute of Jinzhou Medical University, Jinzhou, 121001, China

**Keywords:** lung cancer, ING5, pathogenesis, aggressiveness, prognosis

## Abstract

ING5 can interact with p53, thereby inhibiting cell growth and inducing apoptosis. We found that ING5 overexpression not only inhibited proliferation, migration, and invasion, but also induced G2 arrest, differentiation, autophagy, apoptosis, glycolysis and mitochondrial respiration in lung cancer cells. ING5 transfection up-regulated the expression of Cdc2, ATG13, ATG14, Beclin-1, LC-3B, AIF, cytochrome c, Akt1/2/3, ADFP, PFK-1 and PDPc, while down-regulated the expression of Bcl-2, XIAP, survivin,β-catenin and HXK1. ING5 transfection desensitized cells to the chemotherapy of MG132, paclitaxel, and SAHA, which paralleled with apoptotic alteration. ING5 overexpression suppressed the xenograft tumor growth by inhibiting proliferation and inducing apoptosis. ING5 expression level was significantly higher in normal tissue than that in lung cancer at both protein and mRNA levels. Nuclear ING5 expression was positively correlated with ki-67 expression and cytoplasmic ING5 expression. Cytoplasmic ING5 expression was positively associated with lymph node metastasis, and negatively with age, lymphatic invasion or CPP32 expression. ING5 expression was different in histological classification: squamous cell carcinoma > adenocarcinoma > large cell carcinoma > small cell carcinoma. Taken together, our data suggested that ING5 downregulation might involved in carcinogenesis, growth, and invasion of lung cancer and could be considered as a promising marker to gauge the aggressiveness of lung cancer. It might be employed as a potential target for gene therapy of lung cancer.

## INTRODUCTION

Inhibitor of growth 5 (ING5) belongs to the encoding protein of Class II tumor suppressor gene (TGS) since its inactivation results from frequent genetic and epigenetic alterations [1, 2]. Structurally, it includes LZL (leucine zipper like), NCR (novel conserved region), NLS (nuclear localization signal), and PHD (plant homeo domain) domains from N-terminal to C-terminal. LZL domain has been shown to induce DNA repair, apoptosis and chromatin remodeling. NCR domain can bind to histone acetyl transferase (HAT) complexes during chromatin remodeling and gene expression [3, 4]. ING5 interacts with histone H3K4me3 to form histone H3-ING5-MOZ-MORF- BRPF and H4-ING5-HBO1-JADE HAT complexes [3–7]. The latter promotes DNA replication via the interaction with mini-chromosome maintenance protein because ING5 knockdown completely abolishes DNA synthesis, and HBO1 knockdown increases S-phase cells ratio [8]. ING5 was reported to activate the cyclin-dependent kinase inhibitor p21/waf1 promoter and acetylated ING5 subsequently bound to the promoters of its target apoptotic genes, including Bax and GADD45 [9, 10]. The antiproliferative effect of ING5 depended on its interaction with INCA1 [11]. ING5 enhanced p53 Lys-382 and Lys-120 acetylation for the pathological and physical interaction [9, 10].

ING5 overexpression can decrease colony-forming efficiency, S-phase cell population, and induce apoptosis of RKO cells in a p53-dependent manner [9]. The intact ING5 can inhibit proliferation and induce apoptosis in HSC-3 cells, while two truncated fragments of ING5 (aa 1-184 and 107-226) induce cellular senescence with cyclin E and CDK2 hypoexpression [12]. ING5 overexpression reverses the aggressive phenotypes of gastric cancer cells such as autophagy and proliferation, dedifferentiation, migration, invasion and lamellipodia formation. ING5 overexpression activates both β-catenin and NF-κB pathways in SGC-7901 cells, and promotes the expression of down-stream genes (e.g. *c-myc*, *Cyclin D1*, *survivin*, and *interleukins*). ING5- mediated chemoresistance was closely related to their apoptotic resistance, Akt activation, and the overexpression of chemoresistance-related genes [13]. ING5 deletion was found to initiate carcinogenesis of ameloblastoma and oral cancers [14, 15]. Recently, down-regulated *ING5* mRNA expression and the missense mutations of its LZL and NCR domains were detectable in oral squamous cell carcinoma [16]. The nuclear ING5 was lowly expressed in the tumorigenesis and promoted apoptosis and cell cycle arrest by interacting with p300 and p21 proteins in neck squamous cell carcinoma (HNSCC) [17]. The nuclear to cytoplasmic shift of ING5 protein occurred during colorectal, gastric and HNSCC carcinogenesis and was positively linked to the aggressive behaviors of colorectal and gastric cancers [17–19]. ING5 overexpression suppressed growth, blood supply and lung metastasis of SGC-7901 cells by inhibiting proliferation and enhancing autophagy in xenograft models [13].

Lung cancer is one of the most common malignancies and accounts for more deaths than any other cancers in both men and women worldwide despite the increased survival of cancer patients who receive advanced chemotherapy [20, 21]. In the present study, we observed the effects of ectopic ING5 overexpression on aggressive phenotypes of lung cancer cells, and analyzed the relevant molecular mechanisms. ING5 expression was examined in lung cancers, and compared with their clinicopathological parameters. Finally, we observed the effects and mechanisms of ING5 expression on the growth of lung cancer cells using nude mice model.

## RESULTS

### The effects of *ING5* expression on the phenotypes and their related mechanisms of lung cancer cell lines

**Figure 1:**
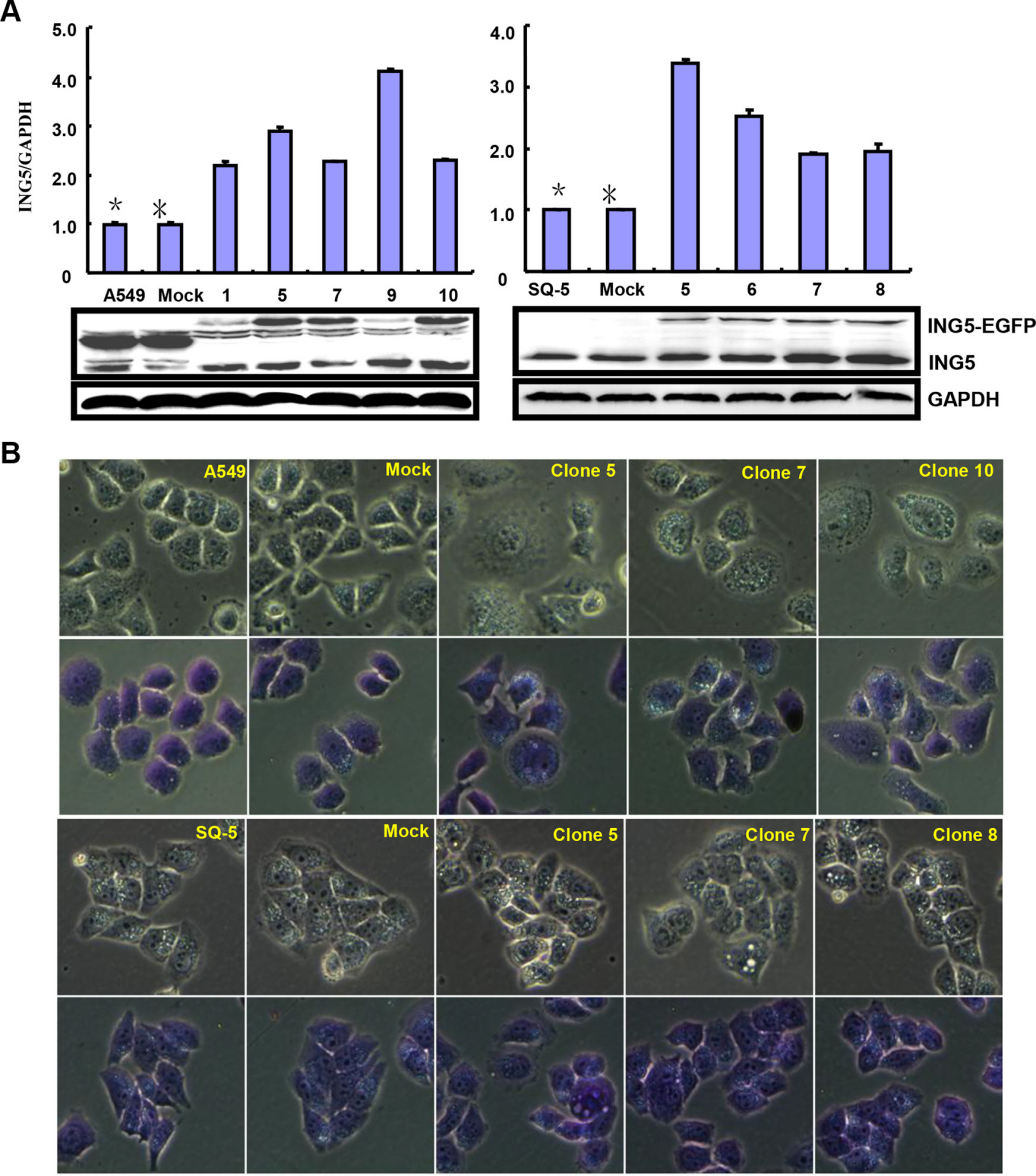

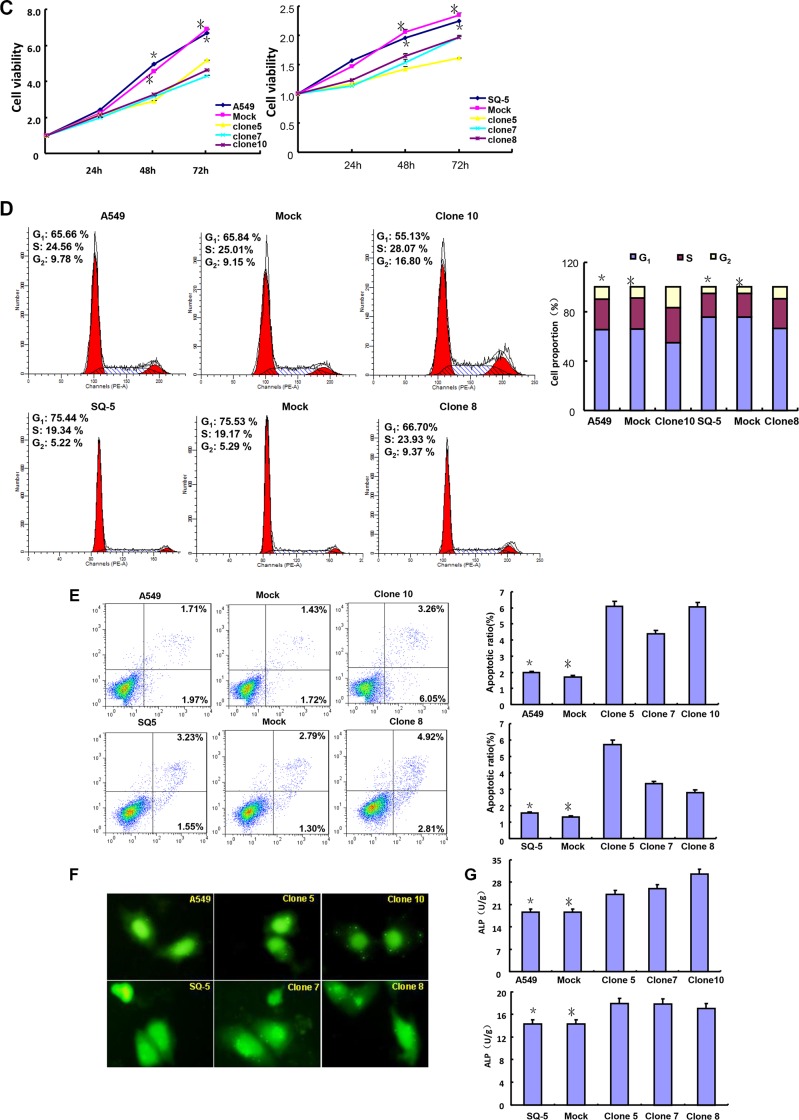

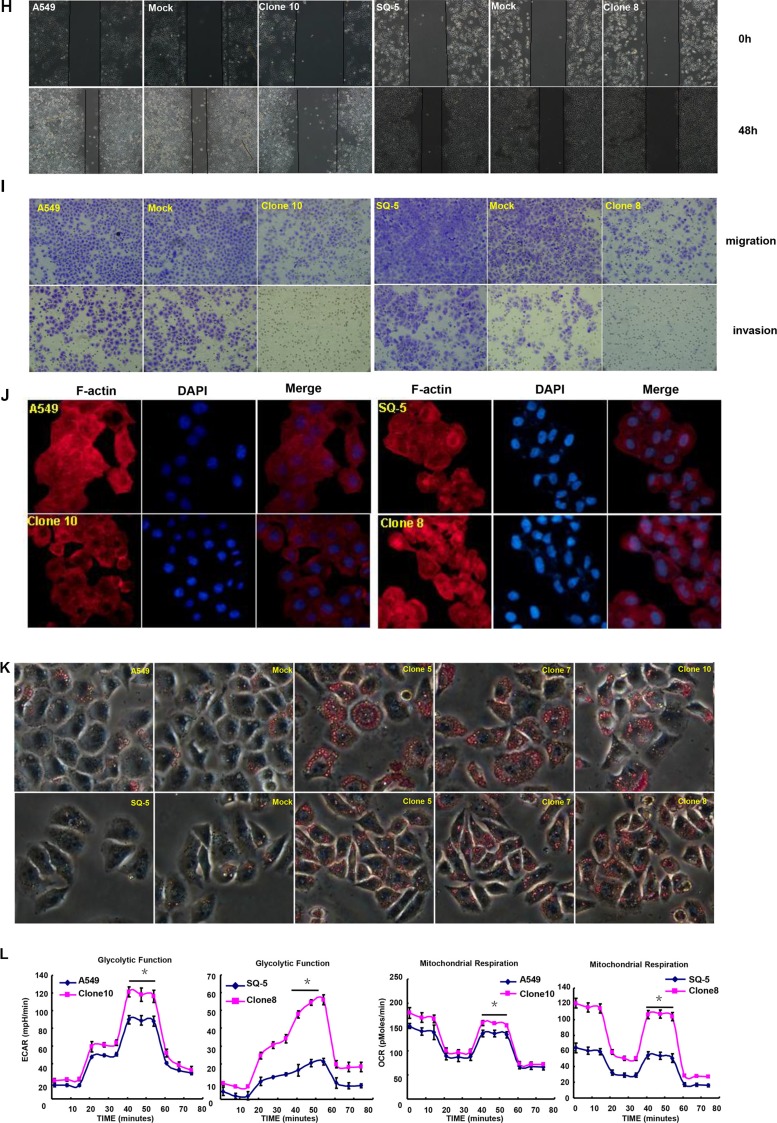

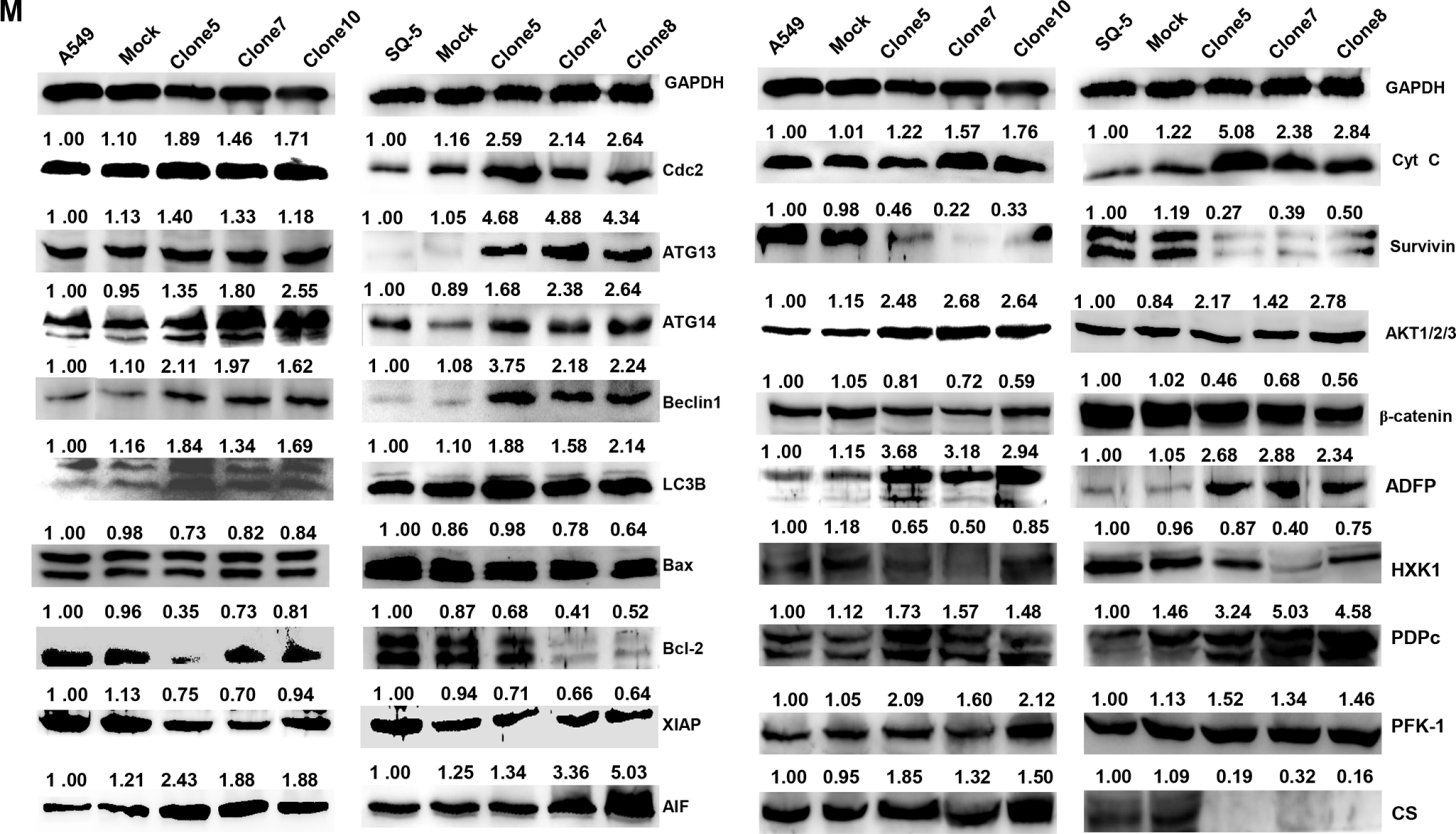
ING5 overexpression represses the aggressive phenotypes of lung cancer cells and causes the alteration in the phenotype- related proteins expression After transfection of pEGFP-N1-*ING5*, *ING5* mRNA and protein were overexpressed in A549 and SQ-5 transfectants by RT-PCR and Western blot (**A**). The transfectants showed mitotic disruption evidenced by contrast microscopy and crystal violet staining (**B**) and a low viability evidenced by CCK-8 (**C**), G_2_ arrest by PI staining (**D**), a high apoptosis by Annexin V (**E**), autophagy by GFP- tagged LC-3B transfection (**F**), and a well differentiation by ALP activity (**G**) in comparison with the control and mock. ING5-overexpressing lung cancer cells had a weaker ability to migrate and invade by wound healing (**H**) and transwell chamber assay (**I**). It was no significant changes in invasive pseudopodia formation after *ING5*-expressing plasmid transfection (**J**). There appeared aberrant fat accumulation by oil red O staining (**K**), a high glycolysis and mitochondrial respiration by metabolism assays (**L**) in ING5 transfectants, compared with the control and mock. The phenotype-related proteins were screened by Western blot (**M**). *p < 0.05, compared with ING5 transfectants. *p < 0.05, compared with ING5 transfectants.

After transfected with pEGFP-N1-*ING5*, A549 (adenocarcinoma) and SQ-5 (squamous carcinoma) cells overexpressed *ING5* at both mRNA and protein levels (Figure 1A, *p* < 0.05). There was a more mitotic disruption by contrast microscopy and crystal violet staining (Figure 1B) and a slower growth evidenced by CCK-8 (Figure 1C, *p* < 0.05) in comparison to the control and mock. Cell cycle analysis indicated that G_2_ arrest in both ING5 transfectants by PI staining (Figure 1D, *p* < 0.05). There was a high level of apoptosis evidenced by Annexin-V (Figure 1E, *p* < 0.05), a high autophagy by pEGFP-tagged LC-3B transfection (Figure 1F) and a good differentiation by ALP activity (Figure 1G, *p* < 0.05) in ING5 transfectants, compared with the control and mock. Additionally, forced ING5 overexpression could suppress migration and invasion by wound healing or transwell chamber assay (Figure 1H and 1I, *p* < 0.05). There was no difference in lamellipodia formation between the control/mock and ING5 transfectants, evidenced by F-actin staining (Figure 1J). ING5-overexpressing lung cancer cells showed aberrant fat accumulation according to oil red O staining (Figure 1K), and enhanced glycolytic and mitochondrial respiration (Figure 1L, *p* < 0.05). Further Western-blotting showed higher expression of Cdc2, ATG13, ATG14, Beclin-1, LC-3B, AIF, cytochrome c, Akt1/2/3, ADFP, PFK-1 and PDPc in A459 and SQ-5 transfectants, but did lower expression of Bcl-2, XIAP, survivin,β-catenin and HXK1 than the control and mock (Figure 1M).

**Figure 2:**
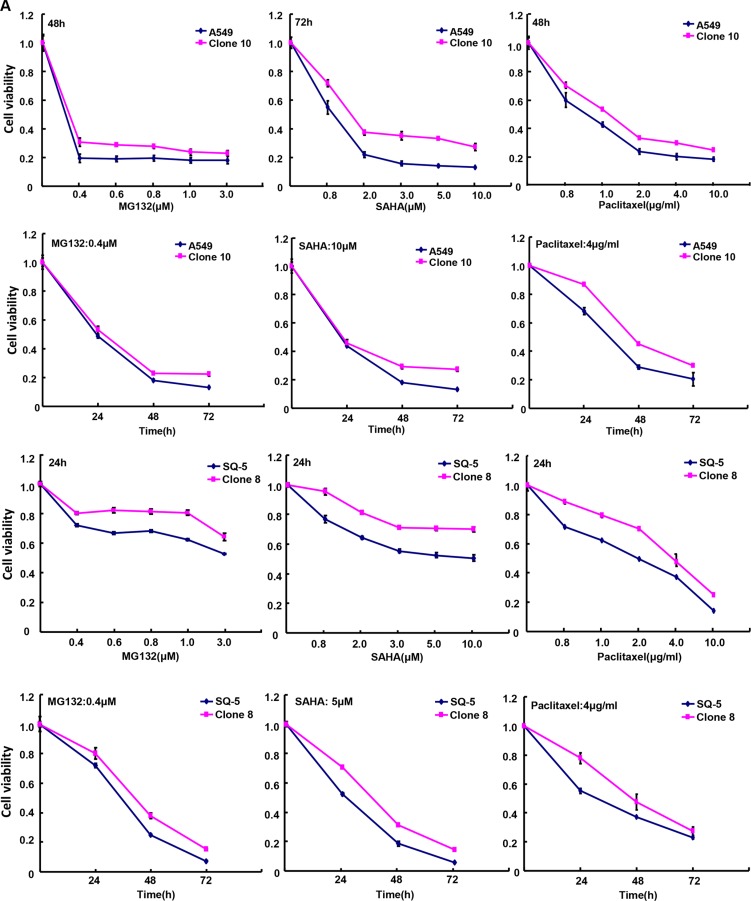

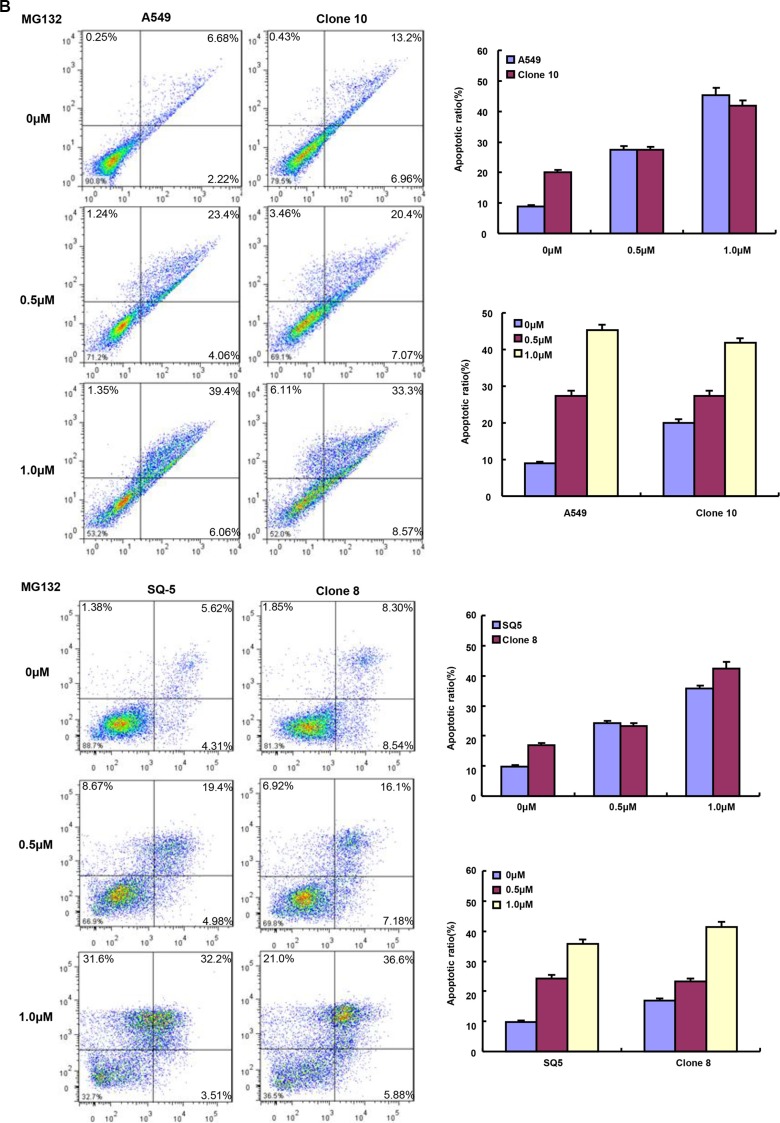

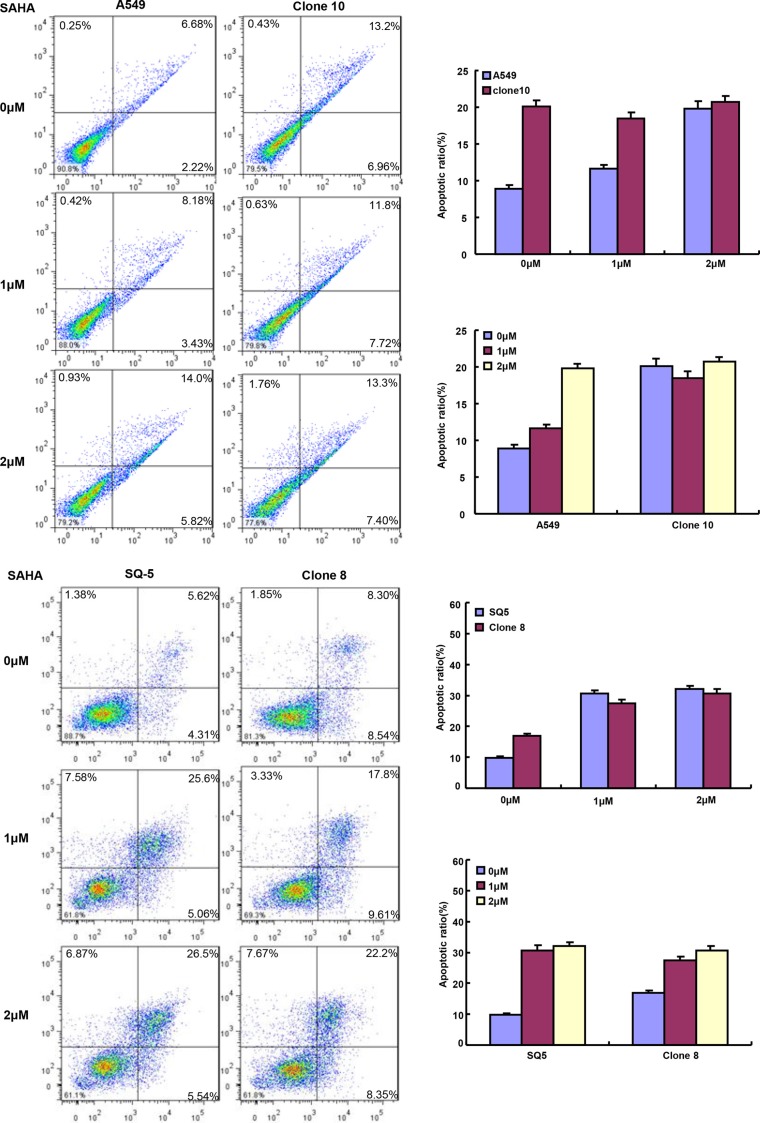

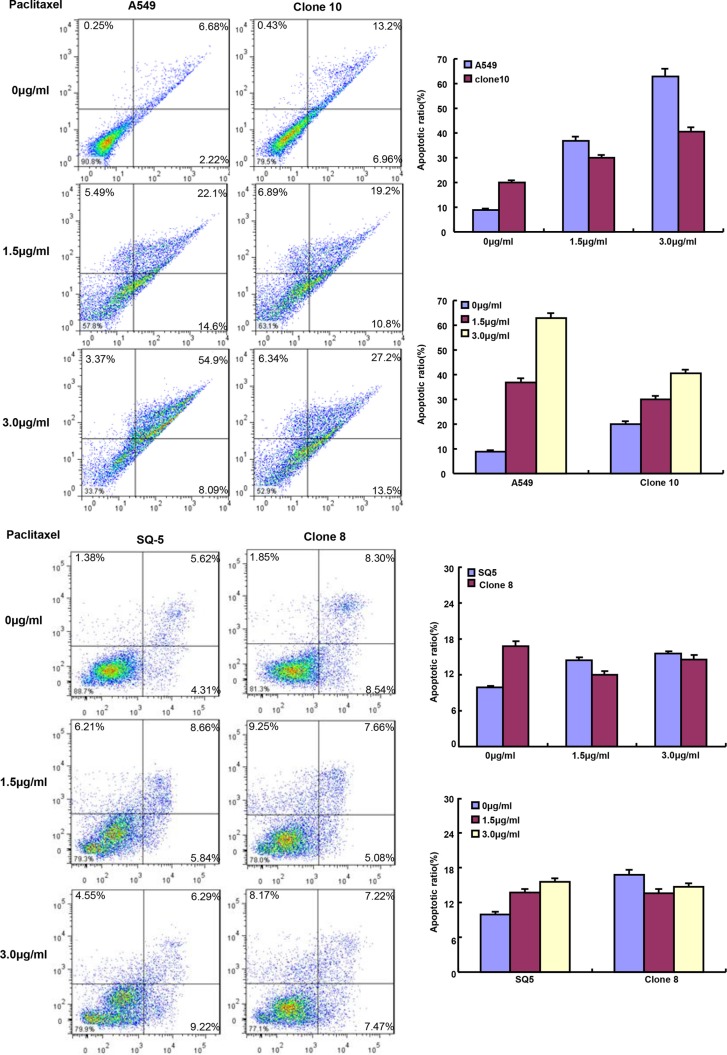
ING5 expression weakened the sensitivity of lung cancer cells to chemotherapeutic agents After treated with MG132, paclitaxel and SAHA, ING5 transfectants showed a higher viability (**A**) and a 1ower apoptotic level (**B**) than the control in both concentration-and time-dependent manners (*p* < 0.05).

**Figure 3:**
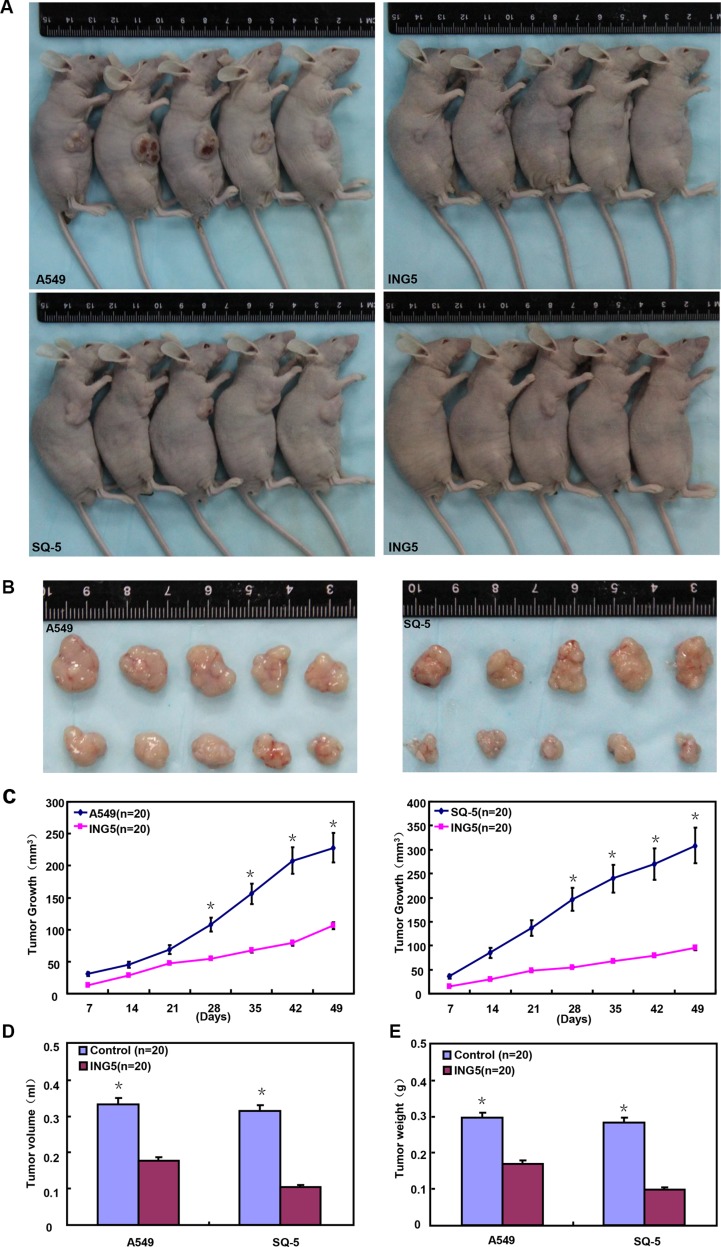

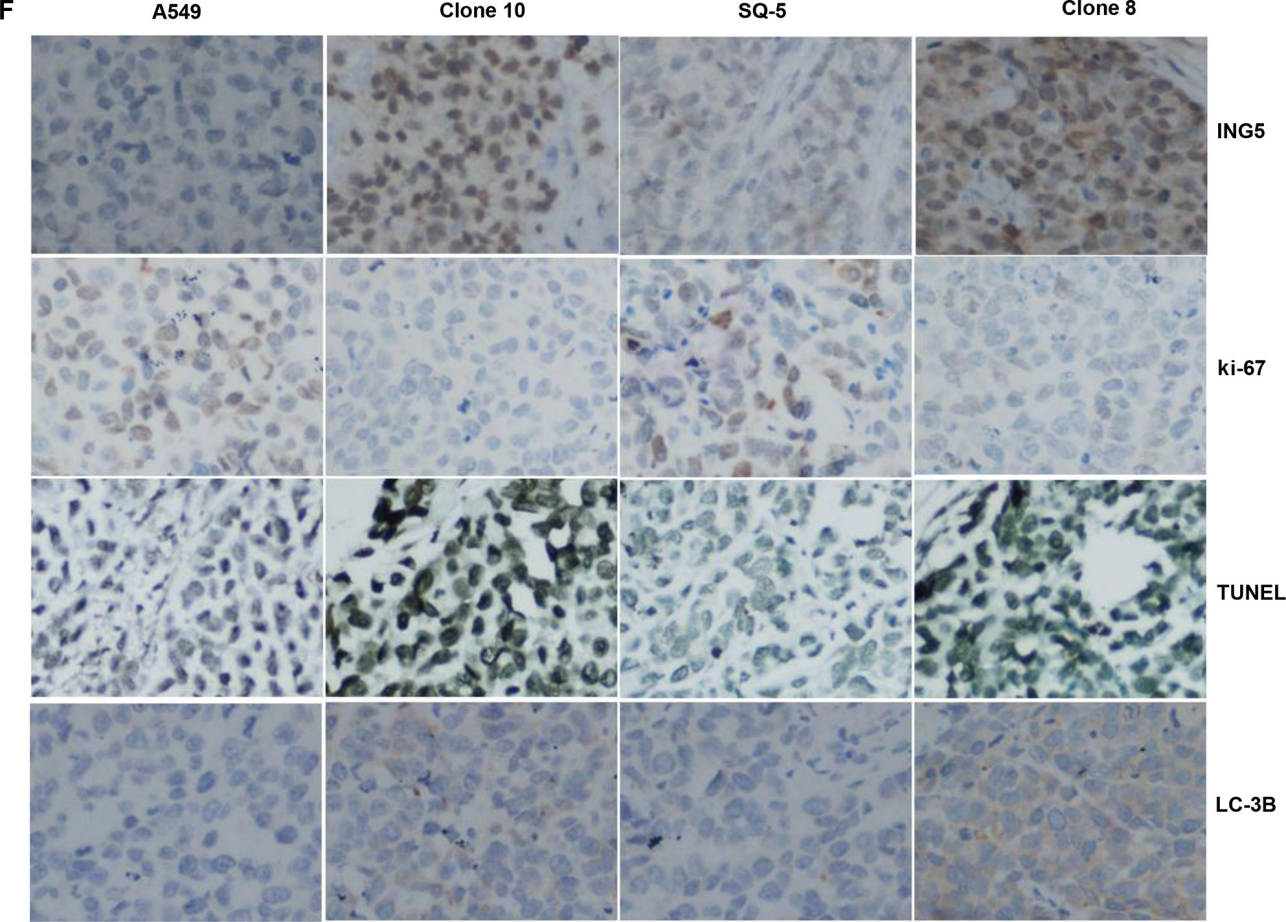
ING5 suppresses the growth of lung cancer cells in the nude mice ING5 transfectant cells showed a slower growth (**A**–**C**), and a lower weight (**D**) and volume (**E**) of xenograft tumors. There were a weaker ki-67 expression, a stronger TUNEL signal and LC-3B expression (**F**) in A549 and SQ-5 transfectants than the control. The data is expressed as mean ± standard deviation; *p < 0.05, compared with ING5 transfectants.

After the exposure to different anti-cancer agents (MG132, paclitaxel, and SAHA), both transfectants showed higher viability and lower apoptosis than those of the control in both time- and dose-dependent manners (Figure 2A and 2B, *p* < 0.05). In the nu/nu mice, ING5 suppressed the xenograft tumor growth by tumor volume and weight (Figure 3A–3E, *p* < 0.05), inhibited proliferation, induced apoptosis and autophagy according to immunohistochemistry and TUNEL (Figure 3F).

### Association of ING5 expression with the clinicopathological parameters of lung cancers

**Figure 4:**
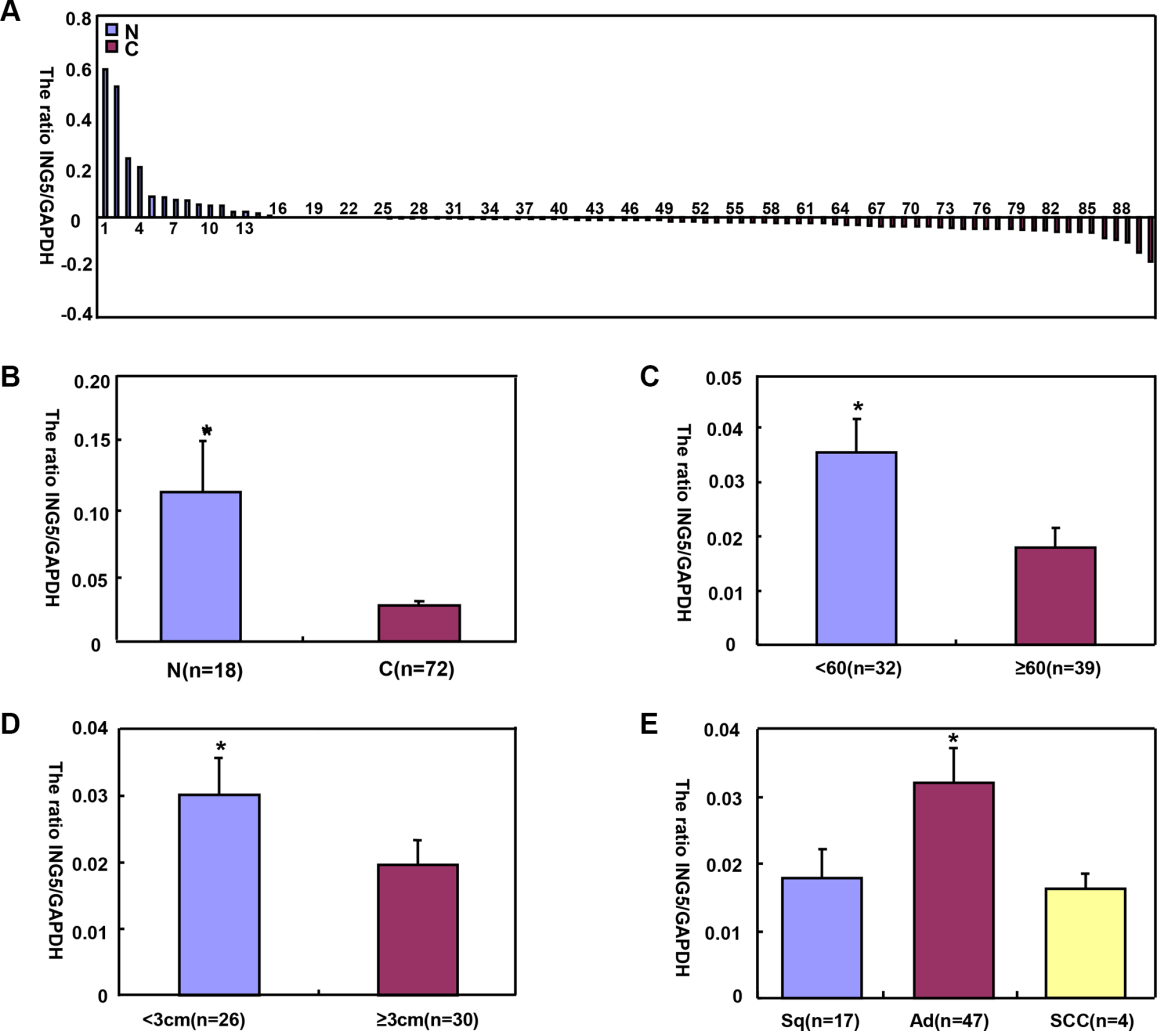
The correlation of *ING5* mRNA expression with clinicopathological features of lung cancers *ING5* mRNA was quantified normal lung tissue (N, *n* = 18) and lung cancers (C, *n* = 72) with GAPDH as an internal control by real-time PCR (**A**). *ING5* mRNA expression level was significantly higher in normal tissue than lung cancer (**B**, *p* < 0.05), and negatively associated with tumor size of lung cancer (**D**) *p* < 0.05). Higher *ING5* mRNA expression was detected in younger patients (< 60 years) with lung cancer than the counterparts (**C**) *p* < 0.05). *ING5* mRNA was overexpressed in adenocarcinoma, in comparison to squamous cell carcinoma and small cell carcinoma (**E,** *p* < 0.05). The data is expressed as mean ± standard error; *p < 0.05.

**Figure 5:**
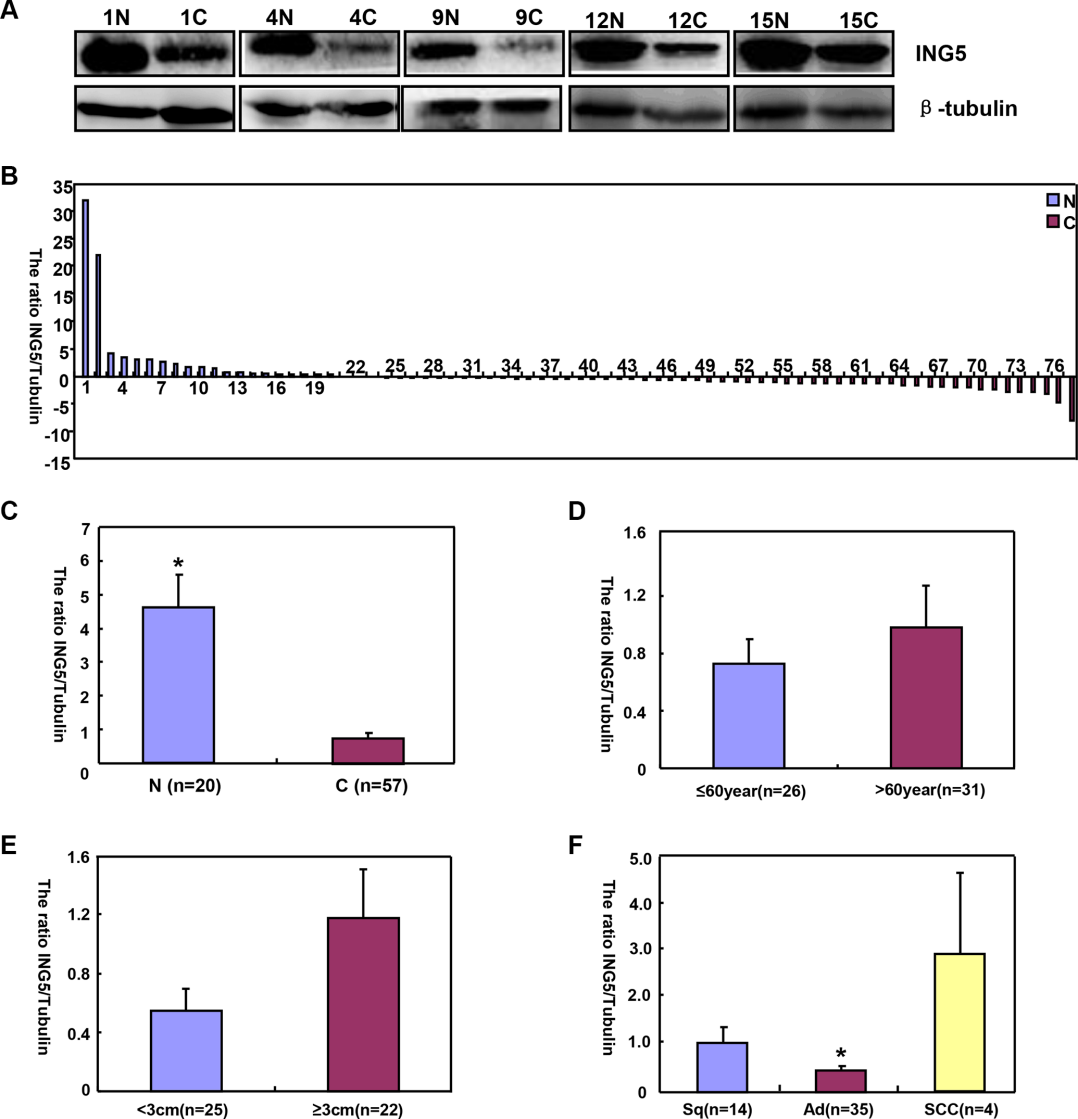
The correlation of ING5 protein expression with clinicopathological features of lung cancers Tissue lysate was loaded and probed with anti-ING5 antibody with β-tubulin as an internal control in normal tissue (N, *n* = 20) and lung cancer (C, *n* = 57) by Western blot. The densitometric analysis showed higher ING5 expression in normal tissue than in lung cancer (**A**–**C**, *p* < 0.05). ING5 expression was not related to age or tumor size (**D** and **E)**, *p* > 0.05). ING5 was hypoexpressed in adenocarcinoma (Ad), in comparison to squamous cell carcinoma and small cell carcinoma (**F**, *p* < 0.05). The data is expressed as mean ± standard error; *p < 0.05.

*ING5* mRNA levels was significantly higher in normal tissue than lung cancer (Figure 4A and 4B, *p* < 0.05), and negatively associated with tumor size of lung cancer (Figure 4D, *p* < 0.05). Higher *ING5* mRNA expression was detected in younger patients (< 60 years) with lung cancer than the counterparts (Figure 4C, *p* < 0.05). *ING5* mRNA was overexpressed in adenocarcinoma (Ad), in comparison to squamous cell carcinoma (Sq) and small cell carcinoma (SCC, Figure 4E, *p* < 0.05). The densitometric analysis showed ING5 protein overexpression in normal tissue, compared with lung cancer (Figure 5A–5C, *p* < 0.05). ING5 expression was not related to age or tumor size of lung cancer (Figure 5D and 5E, *p* > 0.05). ING5 protein was hypoexpressed in Ad, in comparison to Sq and SCC (Figure 5F, *p* < 0.05).

**Figure 6:**
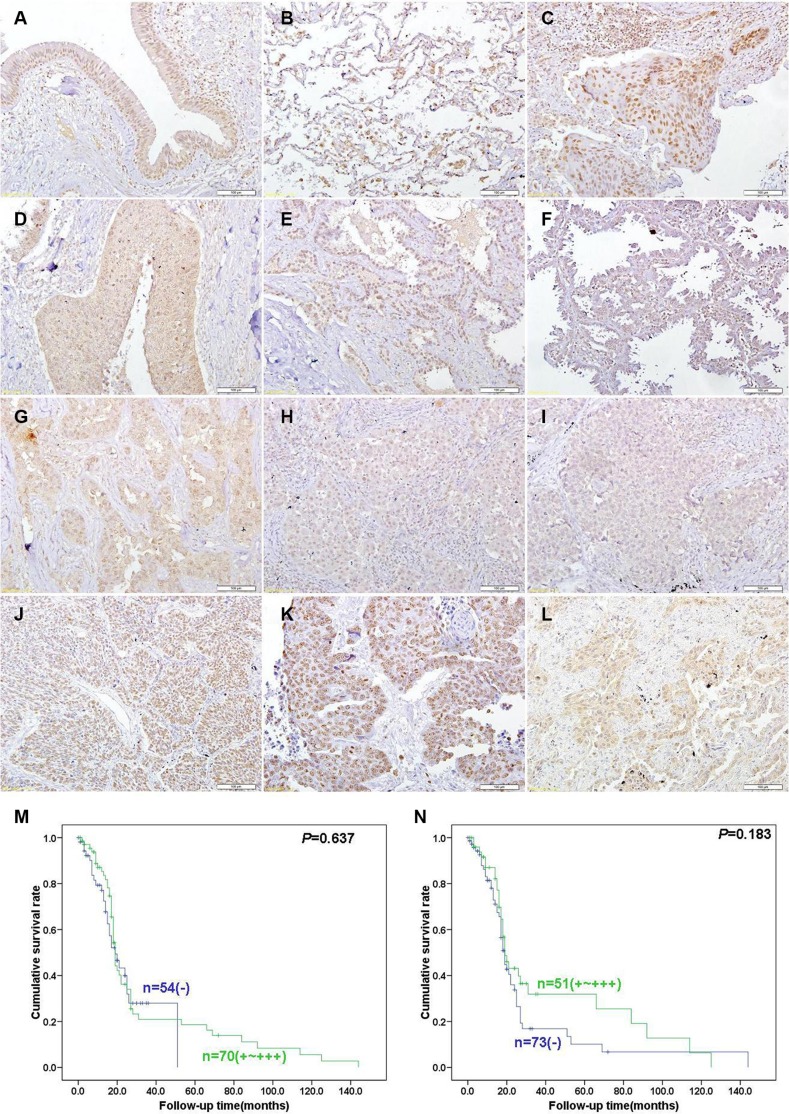
Immunohistochemical staining in lung cancer and adjacent normal lung tissues Note: ING5 is distributed to the nucleus or cytoplasm and Caspase-3 protein is located in the cytoplasm, at the same time p53 and ki-67 is distributed to the nucleus. ING5 was expressed in pseudo-stratified columnar epithelium (**A**), alveolar epithelial cells, macrophage (**B**), squamous cell carcinoma (**C**, **D**), adenocarcinoma (**E**, **F**, **G**), large cell carcinoma (**H**), and small cell carcinoma (**I**). p53 (**J**), ki-67 (**K**) and Caspase-3 (**L**) were strongly expressed in lung cancer. Kaplan-Meier curves relating to the cumulative survival rate of the patients with lung cancer were analyzed according to nuclear (**M**) *p* = 0.637) or cytoplasmic (**N**) *p* = 0.183) ING5 expression status.

ING5 protein was detected in the cytoplasm and nucleus of pseudo-stratified columnar epithelium, alveolar epithelial cells, macrophage, squamous cell carcinoma, adenocarcinoma, large cell carcinoma and squamous cell carcinoma (Figure 6A–6I). p53, ki-67 and Caspase-3 were strongly expressed in lung cancer (Figure 6J–6L). As summarized in Table 1, nuclear ING5 expression was positively correlated with ki-67 expression and cytoplasmic ING5 expression (*p* < 0.05), but no relation was found with sex, age, lymphatic or venous invasion, lymphatic node metastasis, clinicopathological staging, CPP32 or p53 expression (*p* > 0.05). Cytoplasmic ING5 expression was positively associated with lymph node metastasis, and negatively related with age, lymphatic invasion and CPP32 expression (*p* < 0.05), but not correlated with sex, venous invasion, clinicopathological staging, ki-67 or p53 expression (Table 2, *p* > 0.05). Nuclear and cytoplasmic ING5 expression became gradually weaker from Sq, Ad, LCC (large cell carcinoma) to SCC (*p* < 0.05, Tables 1–2). Follow-up information was available on 124 patients with lung cancer for periods ranging from 1 month to 12 years (mean = 20.5 months). Univariate analysis using Kaplan-Meier method indicated that either nuclear or cytoplasmic ING5 expression was no related with the survival rate of the patients with lung cancer (Figure 6M and 6N, *p* > 0.05).

**Table 1:** Relationship between nuclear ING5 expression and clinicopathological parameters of lung cancers

Clinicopathological features	*n*	Nuclear ING5 expression					
		−	+	++	+++	PR (%)	*P* value
Sex							0.452
Female	214	35	65	71	43	83.6	
Male	440	79	141	137	83	82.0	
Age(years)							0.781
< 65	363	57	120	119	67	84.2	
≥ 65	291	57	86	89	59	80.4	
Tumor size (cm)							0.184
< 3	211	31	62	67	51	85.3	
≥ 3	408	56	143	138	71	86.3	
Histological classification							0.001
Squamous cell carcinoma	288	33	102	90	63	88.5	
Adenocarcinoma	317	58	90	110	59	81.7	
Large cell carcinoma	28	13	8	6	1	53.5	
Small cell carcinoma	21	12	4	2	3	42.9	
Lymphatic invasion							0.752
−	488	84	154	156	94	82.8	
+	159	29	50	51	29	81.8	
Venous invasion							0.223
−	114	49	12	22	31	57.0	
+	18	11	0	4	3	38.9	
Lymph node metastasis							0.512
−	395	76	119	125	75	80.8	
+	250	38	82	82	48	84.8	
UICC staging							0.844
I–II	506	86	158	167	95	83.0	
III–IV	144	26	47	41	30	81.9	
ki-67 expression							0.042
−	60	31	6	12	11	48.3	
+ ~ +++	70	27	6	14	23	61.4	
CPP32 expression							0.770
−	30	15	4	4	7	50.0	
+ ~ +++	101	44	8	22	27	56.4	
p53 expression							0.462
−	72	29	10	16	17	59.7	
+ ~ +++	60	31	2	10	17	48.3	
Cytoplasmic ING5 expression							< 0.001
−	101	50	20	14	17	50.5	
+ ~ +++	553	64	186	194	109	88.4	

Abbreviations, PR, positive rate; UICC, Union Internationale Contre le Cancer.

**Table 2:** Relationship between cytoplasmic ING5 expression and clinicopathological parameters of lung cancers

Clinicopathological features	*n*	Cytoplasmic ING5 expression					
		−	+	++	+++	PR (%)	*P* value
Sex							0.253
Female	214	40	60	85	29	81.3	
Male	440	61	128	188	63	86.1	
Age(years)							0.025
< 65	363	43	109	153	58	88.1	
≥ 65	291	58	79	120	34	80.0	
Tumor size(cm)							0.104
< 3	211	47	56	79	29	77.7	
≥ 3	408	53	125	182	48	87.0	
Histological classification							< 0.001
Squamous cell carcinoma	288	22	95	122	49	92.4	
Adenocarcinoma	317	55	82	142	38	82.6	
Large cell carcinoma	28	12	7	7	2	57.1	
Small cell carcinoma	21	12	4	2	3	42.8	
Lymphatic invasion							0.044
−	488	65	143	207	73	86.7	
+	159	34	43	65	17	78.6	
Venous invasion							0.149
−	114	68	9	24	13	40.4	
+	18	7	2	7	2	61.1	
Lymph node metastasis							0.046
−	395	76	107	160	52	84.9	
+	250	25	78	109	38	90.0	
UICC staging							0.109
I–II	506	69	146	223	68	88.9	
III–IV	144	31	42	49	22	78.4	
ki-67 expression							0.370
−	60	37	5	14	4	38.3	
+ ~ +++	70	38	6	16	10	45.7	
CPP32 expression							0.005
−	30	24	2	3	1	80.0	
+ ~ +++	101	51	9	28	13	49.5	
p53 expression							0.134
−	72	44	7	16	5	38.9	
+ ~ +++	60	31	4	15	10	48.3	

Abbreviations, PR, positive rate; UICC, Union Internationale Contre le Cancer.

## DISCUSSION

Down-regulated expression of nuclear ING5 protein was observed in oral Sq, HNSCC and colorectal cancer (CRC) respectively [16–19]. Cytoplasmic translocation of ING5 had impact on the carcinogenesis of HNSCC and CRCs and cytoplasmic ING5 was positively correlated with the aggressive behaviors of CRC and gastric cancers [17, 18]. Here, *ING5* mRNA and protein were found to decrease in lung cancer, compared with those of normal lung tissues, indicating that its deregulation might promote lung tumorigenesis. ING5 expression became gradually weaker from Sq, Ad, LCC to SCC, suggesting that ING5 might be employed as a marker to differentiate the histological subtypes of lung cancer. Consistent with another report [18], cytoplasmic ING5 was negatively associated with lymphatic invasion of lung cancer, which might be attributable to no wide and careful application of D2-40 immunostaining in the pathological diagnosis. The positive correlation between cytoplasmic ING5 and lymph node metastasis suggested that it could be regarded as a marker for aggressiveness, supported by lower CPP32 (a apoptotic marker) in the cases with cytoplasmic ING5 positive. The positive relationship between nuclear ING5 and ki-67 (a proliferative marker) expression might be due to a feedback overexpression of nuclear ING5 in lung cancer.

Since pulmonary Ad and Sq histologically rank the first and two, we chose A549 and SQ-5 for cell functional experiments. It was that ING5 overexpression induced apoptosis, autophagy and differentiation, and inhibited the proliferation, cell mitosis and cell cycle progression, migration and invasion. In tumor-bearing nude mouse model, ING5 was demonstrated to suppress the tumor growth by inducing apoptosis and autophagy, and decreasing proliferative ability. However, it was worth noting that ING5 might induce chemoresistance, which was positively linked to apoptotic resistance, in line with a previous report [13]. In other words, ING5 up-regulated the apoptotic level of lung cancer cells, but had no ability to induce their apoptotic level against chemodrugs. In combination with the findings in gastric cancer cells, it should be possible and practicable to employ ING5 as a potential target for gene therapy of malignancies if its chemoresistance could be dislodged. Reportedly, ING5 inhibited cancer aggressiveness of lung cancer via preventing epithelial to mesenchymal transition [22], but it was regret that the phenomenon was not observed although we utilized the same cancer cell, A549.

In lung cancer cells, ectopic ING5 expression caused G_2_ phase arrest and suppressed proliferation, while the overexpression of Cdc2 protein might be attributed to the higher proportion of cells in G_2_ phase [23]. It is well known that Bax interacts with Bcl-2, promotes cytochrome c release, and activates the endogenous apoptosis pathway, which is abolished by survivin [24]. AIF transfers from the mitochondria to the cytoplasm, and then enters the nucleus, causing nuclear DNA aggregate and fractures [25]. ING5 overexpression up-regulated the expression of pro-apoptotic proteins AIF and cytochrome c, and down-regulated the expression of anti-apoptotic proteins Bcl-2, XIAP and survivin, accounting for its apoptosis-induced role in lung cancer cells by mitochondrial pathway. Autophagy might wrap organelles and proteins in autophagic classical pathway by the complexes, including ATG13, ATG14, Beclin-1 and LC-3B [26, 27]. Here, it was found that ING5 overexpression *in vivo* and vitro induced the autophagy of lung cancer cells with ATG13, ATG14 and Beclin-1 overexpression, indicating that ING5-induced autophagy was dependent on Beclin-1 and belonged to canonical pathway.

Hexokinase I (HXKI) utilizes Mg^**2**+^-ATP as a phosphoryl donor to catalyze the first step of intracellular glucose metabolism, the conversion of glucose to glucose-6-phosphate [28]. Phospho- fructokinases 1(PFK-1) is a regulatory glycolytic enzyme that converts fructose 6- phosphate and ATP into fructose 1, 6-bisphosphate [29]. Pyruvate dehydrogenase phosphatase c (PDP c) is localized within the mitochondrial matrix and efficiently dephosphorylates all three phosphorylation sites located on α chain of the E1 component, which simultaneously activates pyruvate dehydrogenase to convert pyruvate to acetyl-CoA for utilization in the Kreb's Cycle [30]. Adipophilin (ADFP) is a ubiquitous component of lipid droplets and its overexpression is a useful marker for pathologies characterized by increased lipid droplet accumulation [31]. Here, we for the first time found that ING5 overexpression increased glycolysis and subsequent aerobic oxidation, which is closely linked to PFK-1 and PDPc overexpression. Additionally, aberrant fat accumulation in ING5 transfectants might be attributable to the up-regulatory ADFP expression.

In summary, down-regulation expression of ING5 protein could be involved in lung carcinogenesis and closely linked to the histogenesis of non-small cell lung cancers. ING5 overexpression might suppress the proliferation, migration and invasion, induce apoptosis, autophagy, and differentiation, and mediate chemotherapeutic resistance of lung cancer cells. Therefore, it has to be possible to employ ING5 as a target of gene therapy for lung cancer if its chemoresistance could be overcome. We for the first time reported that ING5 might induce glucose catabolism and aberrant fat deposition in lung cancer cells.

## MATERIALS AND METHODS

### Cell culture and transfection

Lung cancer cell lines, A549 and SQ-5 came from Japanese Physical and Chemical Institute. They were grown in RPMI 1640 medium supplemented with 10% fetal bovine serum (FBS), 100 units/ml penicillin, and 100 μg/ml streptomycin in an atmosphere of 5% CO_2_ at 37°C. The cells were collected by centrifugation and rinsed with phosphate buffered saline (PBS, pH 7.2) for RNA and protein extract.

### Crystal violet and oil red O staining

To observe the morpholocial appearance, we fixed cells in 100% methanol for 20 min and stained with crystal violet. At aim to identify the fat acculumation, the cells were fixed in ice cold 10% formalin for 30 min and rinsed with PBS for three times. After that, the plates were incubated with pre-warmed Oil Red O solution for 15 min.

### Proliferation assay

Cell Counting Kit-8 (CCK-8) was employed to determine cell viability. In brief, 2.0 × 10^3^ cells/well were seeded on 96-well plate and allowed to adhere. At different time points, 10 μL of CCK-8 solution was added into each well and the plates were incubated for 3 h in the incubator, and then measured at 450 nm.

### Cell cycle analysis

The cells were trypsinized, collected, washed by PBS twice and fixed in cold 10 mL ethanol for more than 2 h at −20°C. And then, cells were washed by PBS twice and incubated with 1 mL RNase (0.25 mg/mL) at 37°C for 1 h. The cells were pelleted and resuspended in propidium iodide (PI) at a concentration of 50 μg/mL and incubated at 4°C in the dark for 30 min. Finally, flow cytometry was employed to examine PI signal.

### Apoptosis assay by flow cytometry

Flow cytometry was performed with PI and FITC-labeled Annexin V (Beyotime, China) to detect phosphatidylserine externalization as an endpoint indicator of apoptosis as the manufacturer's instructions describe.

### Immunofluorescence

Cells were grown on glass coverslips, washed twice with PBS, fixed with 4% formaldehyde for 10 min at room temperature, and permeabilized with 0.25% Triton X-100 for 10 min at room temperature. After washing with PBS, cells were incubated overnight at 4°C with the phalloidin (Sigma, USA). Alternatively, the sections were mounted with DAPI (KeyGEN). Finally, the microphotography was performed under fluorescence microscopy.

### Cell migration and invasion assays

For the invasion assay, 2.5 × 10^5^ cells were resuspended in serum-free RPMI 1640, and seeded in the matrigel-coated insert on the top portion of the chamber. The lower compartment of the chamber contained 10% v/v FBS as a chemoattractant. After incubated at 37°C and 5% CO_2_ for 24 hours, cells on the membrane were scrubbed, washed with PBS, fixed in 100% methanol and stained with crystal violet. For the migration assay, the procedures were the same as above excluding the control-membrane insert. To confirm the migration assay, wound healing assay was performed as well.

### Metabolism assays

The cells were plated at a density of 12,000 cells per well into XF96 plates and allowed to adhere. Oxygen consumption rates and extracellular acidification rates were measured in XF media (DMEM containing either 10 mM or 15 mM glucose, 2 mM L-glutamine, and 1 mM sodium pyruvate) under basal conditions and in response to mitochondrial inhibitors, 1 mM oligomycin and/or 100 nM rotenone + 1 mM antimycin A on the XF-24 Extracellular Flux Analyzers (Seahorse Bioscience).

### Subjects

Between January 1993 and December 2014, 654 patients underwent surgical resection at The First Affiliated Hospital of Jinzhou Medical University. These cancer tissues were fixed in 4% neutralized formaldehyde, embedded in paraffin and sectioned at 4 μm. The sections were stained with hematoxylin and eosin (HE) to confirm their histological diagnosis according to World Health Organization criteria [32]. The staging for each lung cancer was evaluated according to the Union Internationale Contre le Cancer (UICC) system for the extent of tumor spread [33]. The frozen lung cancer (*n* = 72) and normal tissue (*n* = 20) samples were freshly obtained from Department of Thoaric Surgery, The First Affiliated Hospital of China Medical University (Shenyang). The samples were frozen immediately in liquid nitrogen and stored at −80°C until being used. Informed consent was obtained from all subjects and the study was approved by the Ethics Committee of Jinzhou Medical University and China Medical University.

### Xenograft models

BALB/c nude (nu/nu) mice of 6–8 weeks were bred and used for implantation. The animals were maintained under specific pathogen-free conditions, and food and water were supplied ad libitum. Housing and all procedures involving animals were performed according to protocols approved in compliance with the Committee for Animal Experiments Guidelines on Animal welfare of Jinzhou Medical University. Subcutaneous xenografts were established by injection of 1 × 10^6^ tumor cells/mouse to the axilla (*n* = 20/group). Tumor growth was then monitored for 49 days. Until the end of the experiment, mice were anesthetized, photographed, and sacrificed. The tissues recovered were subjected to further analysis. For each tumor, measurements were made using calipers, and tumor volumes were calculated as follows: length × width × depth × 0.52. The part of tumors were subsequently fixed in 4% paraformaldehyde for 24 h, and then embedded in paraffin.

### Real-time reverse transcriptase-polymerase chain reaction (RT-PCR)

Total RNA was extracted from tissue and cells using QIAGEN RNeasy mini kit and subsequently subjected to cDNA synthesis using Avian Myeloblastosis Virus transcriptase and random primer (Takara, Otsu, Japan). We carried out real-time RT-PCR using SYBR Premix Ex Taq^TM^ II kit. According to the Genbank (NM_032329.4), oligonucleotide primers for *ING5* were designed as follows: forward: 5′- GGGAGATGATTGGCTGTG-3′ and reverse: 5′-CCTTTGGGTT TCGTGGTA-3′ (614-759, 146bp). The primers for the internal control, *GAPDH*, were forward: F: 5′-CAATGACCCCTTCATTGACC-3′ and reverse: 5′- TGGAAGATGGTGATGGGATT-3′ (201- 335, 135bp; NM_ 002046.3).

### Western blot

The denatured protein was separated on a sodium lauryl sulfate (SDS) -polyacrylamide gel (12% acrylamide) and transferred to Hybond membrane, which was then blocked overnight in 5% milk in tris buffered saline with Tween 20 (TBST). For immunoblotting, the membrane was incubated with primary antibody for 1 h (Supplementary Table 1). Then, it was rinsed by TBST and incubated with IgG conjugated to horseradish peroxidase for 1 h. Bands were visualized with X-ray film by ECL-Plus detection reagents (Santa cruz). The densitometric quantification was performed with a GAPDH or β-tubulin control using Scion Image software.

### Tissue microarray (TMA) and immunohistochemistry (IHC)

Representative areas of solid tumors were identified in HE stained sections of the selected tumor cases and a 2 mm-in-diameter tissue core per donor block was punched out and transferred to a recipient block with a maximum of 48 cores using a Tissue Microarrayer (AZUMAYA KIN-1, Tokyo, Japan). IHC was performed using intermittent irradiation in a microwave oven as described previously [34]. After each treatment, the slides were washed with TBST three times for 1 min. After counterstained with Mayer's hematoxylin, the sections were dehydrated, cleared and mounted. Omission of the primary antibody was used as a negative control.

We considered the nuclear or cytoplasmic distribution of ING5 protein for statistical analysis in lung cancer. One hundred cells were randomly selected and counted from 5 representative fields of each section blindly by two independent observers (Zhao S and Zheng HC). The inconsistent data were confirmed by both persons until final agreements were reached. The expression positivity was graded and counted as follows: 0 = negative; 1 = 1–50%; 2 = 50–74%; 3 ≥ 75%. The staining intensity score was graded as follows: 1 = weak; 2 = intermediate; and 3 = strong. The scores for ING5 positivity and staining intensity were multiplied to obtain a final score, which determines their expression as (− = 0; + = 1–2; ++ = 3–5; +++ = 6–9).

### Terminal digoxigenin-labeled dUTP nick-end labeling (TUNEL)

Cell apoptosis was assessed using TUENL, a method that is based on the specific binding O-TdT to the 3-OH ends of DNA, ensuring the synthesis of a polydeoxynucleotide polymer. For this purpose, ApopTag Plus Peroxidase *In Situ* Apoptosis Detection Kit (Chemicon) was employed according to the recommendation. Omission of the working strength TdT enzyme was considered as a negative control.

### Statistical analysis

Statistical evaluation was performed using Spearman correlation test to analyze the rank data and using Mann-Whitney U to differentiate the means of different groups. Kaplan-Meier survival plots were generated and comparisons between survival curves were made with the log-rank statistic. *p* < 0.05 was considered as statistically significant. SPSS 10.0 software was employed to analyze all data.

## SUPPLEMENTARY MATERIALS


