# Supplementary material for: The down-regulated ING5 expression in lung cancer: A potential target of gene therapy
Source: Oncotarget. 2016 Jul 9;7(34):54596–615. doi: 10.18632/oncotarget.10519 (PMC5342367; doi:10.18632/oncotarget.10519)
Supplement: Supplementary file 1 [file oncotarget-07-54596-s001.pdf]

The down-regulated ING5 expression in lung cancer: A potential target of gene therapy

Supplementary Materials

Supplementary Table S1:

Num	Antibody	Species	Dilution	Company	Code number
1	Cdc2 p34 (B-6)	mouse	1:500	Santa cruz	sc-8395
2	ATG13 (D4P1K)	rabbit	1:500	CST	13273
3	ATG14	rabbit	1:500	CST	5504
4	Beclin1	rabbit	1:1500	Abcam	ab55878
5	LC3B	rabbit	1:500	CST	3868
6	Bax (B-9)	mouse	1:500	Santa cruz	sc-7480
7	Bcl-2 (C 21)	rabbit	1:500	Santa cruz	sc-783
8	XIAP (H-202)	rabbit	1:500	Santa cruz	sc-11426
9	AIF (E-1)	mouse	1:700	Santa cruz	sc-13116
10	cytochrome c (C-20)	goat	1:500	Santa cruz	sc-8385
11	Survivin(C-6)	mouse	1:500	Santa cruz	sc-374616
12	Akt1/2/3 (H-136)	rabbit	1:500	Santa cruz	sc-8312
13	β-catenin(C-18)	goat	1:500	Santa cruz	sc-1496
14	ADFP	rabbit	1:2000	Abcam	ab52355
15	HXK1(N-19)	goat	1:500	Santa cruz	sc-6517
16	PDPc(D-11)	mouse	1:500	Santa cruz	sc-398117
17	PFK-1(K-15)	goat	1:500	Santa cruz	sc-31712
18	Citrate synthase(P-20)	goat	1:500	Santa cruz	sc-242444
19	GAPDH	rabbit	1:2000	Santa cruz	10494-1-Ap
20	ING5	rabbit	1:1500	Proteintech	10665-1-Ap
